# Predicting blood transfusion demand in intensive care patients after surgery by comparative analysis of temporally extended data selection

**DOI:** 10.1186/s12911-024-02800-z

**Published:** 2024-12-18

**Authors:** Seyedmostafa Sheikhalishahi, Sebastian Goss, Lea K. Seidlmayer, Sarra Zaghdoudi, Ludwig C. Hinske, Mathias Kaspar

**Affiliations:** 1 Digital Medicine, University Hospital of Augsburg, Augsburg, Germany; 2 Internal Medicine I, Cardiology, University Hospital of Augsburg, Augsburg, Germany

**Keywords:** Machine learning, Blood transfusion, Intensive care unit, Surgical patients, Predictive modeling, XGBoost

## Abstract

**Background:**

Blood transfusion (BT) is a critical aspect of medical care for surgical patients in the Intensive Care Unit (ICU). Timely and accurate identification of BT needs can enhance patient outcomes and healthcare resource management.

**Methods:**

This study aims to determine whether a machine learning (ML) model can be trained to predict the need for blood transfusion (BT) in patients on the ICU after a wide range of surgeries, utilizing only data from the ICU.

**Results:**

This retrospective study analyzed data from 9,118 surgical ICU patients from the Amsterdam University Medical Centers database (UMCdb). The study included a primary analysis using data from 6 h before ICU admission up to 1, 2, 3, and 6 h after admission, and a secondary analysis using only the data from 6 h before ICU admission and only the data from the first hour after admission. The model integrated 32 relevant clinical variables and compared the performance of XGBoost and logistic regression (LR) algorithms.

**Conclusions:**

The model demonstrated an effective BT prediction, with XGBoost outperforming LR, particularly for a 12-hour prediction window. Notable differences in patient characteristics were observed among those who received BT and those who did not receive BT. The study establishes the feasibility of using ML for the prediction of BT in surgical ICU patients. It underlines the potential of ML models as decision support tools in healthcare, enabling early identification of BT needs.

**Supplementary Information:**

The online version contains supplementary material available at 10.1186/s12911-024-02800-z.

## Background

The need for a red blood cell transfusion (BT) is one of the most important complications of surgery [1]. Preparation for a BT is time-consuming, since a BT requires the determination of the blood group and the subsequent examination and preparation of the donor blood. Furthermore, patients requiring BT after surgery often stay longer in the hospital than patients without BT [2]. Thus, it is of great importance to plan a hospital stay as thoroughly as possible, for financial and organizational reasons. Subsequently, for improved planning it would be beneficial to find and optimize prognostic markers that can predict the need for BT [3].

There are already several clinical risk scores that attempt to assess the risk of requiring BT, e.g., for cardiopulmonary bypass and cardiac surgery. However, all scores have been created solely for a specific disease and often for patients in a specific clinical situation. Thus, their usability for the general patient population is limited [4, 5].

There have also been machine learning (ML) models developed to identify the need for BT; mostly perioperatively. Some studies focus on the prediction of the required blood volume [6–8]. Most of these studies provide models for intra or perioperative prediction of BT for specific surgery types, like for spinal surgery [9], pelvic fracture patients [10], cardiac surgery [11, 12], and gastric cancer [13]. There are only few studies targeting BT predictions from the perspective of an Intensive Care Unit (ICU) stay, which focus on gastrointestinal bleeding [14, 15] or cardiothoracic surgery [11]. Only two studies had a broader approach by targeting any kind of surgery in a perioperative setting [16] or all patients after hospital admission [17].

None of the studies assessed the need for BT after any kind of surgery from an ICU perspective, particularly not under consideration of an increasing large amount of data available over time on the ICU.

Therefore, this study aims to determine whether a machine learning (ML) model can be trained to predict the need for blood transfusion (BT) in patients on the ICU after a wide range of surgeries, utilizing only data measured after and shortly before ICU admission.

The focus is to provide an early-stage decision support that improves over time to identify the need for BT during and after ICU admission.

## Methods

### Study design and database description

In this retrospective study, we developed and validated an ML model to predict BT requirements for surgical patients following their admission to ICU. Data was extracted from the Amsterdam University Medical Centers database (UMCdb) version 1.0.2, including 23,106 admissions of 20,109 individual patients from 2003 to 2016. The database provides various types of data, including demographic data, data from patient monitors and life support devices (captured at a frequency of up to one measurement per minute), laboratory measurements, clinical observations and scores, medical procedures, medical tasks, medications, fluid balance, diagnosis groups, and clinical outcomes [18]. During model training and evaluation, we classified outcomes as follows:

- Positive Outcome: Patients who required a blood transfusion during ICU admission after surgery.
- Negative Outcome: Patients who did not require a blood transfusion during ICU admission after surgery.

These classifications were used to train and evaluate the machine learning model, ensuring that the predictions accurately reflect the necessity of blood transfusion in the given patient cohort.

## Cohort selection

We included adult patients, aged 18 or older, who were admitted to the ICU primarily due to undergoing a single surgical procedure. In scenarios where patients had multiple admissions to the ICU, their initial admission was considered for analysis. Additionally, patients with a length of stay of less than 12 h in ICU were excluded.

## Outcome definition and experimental setup

**Fig. 1:**
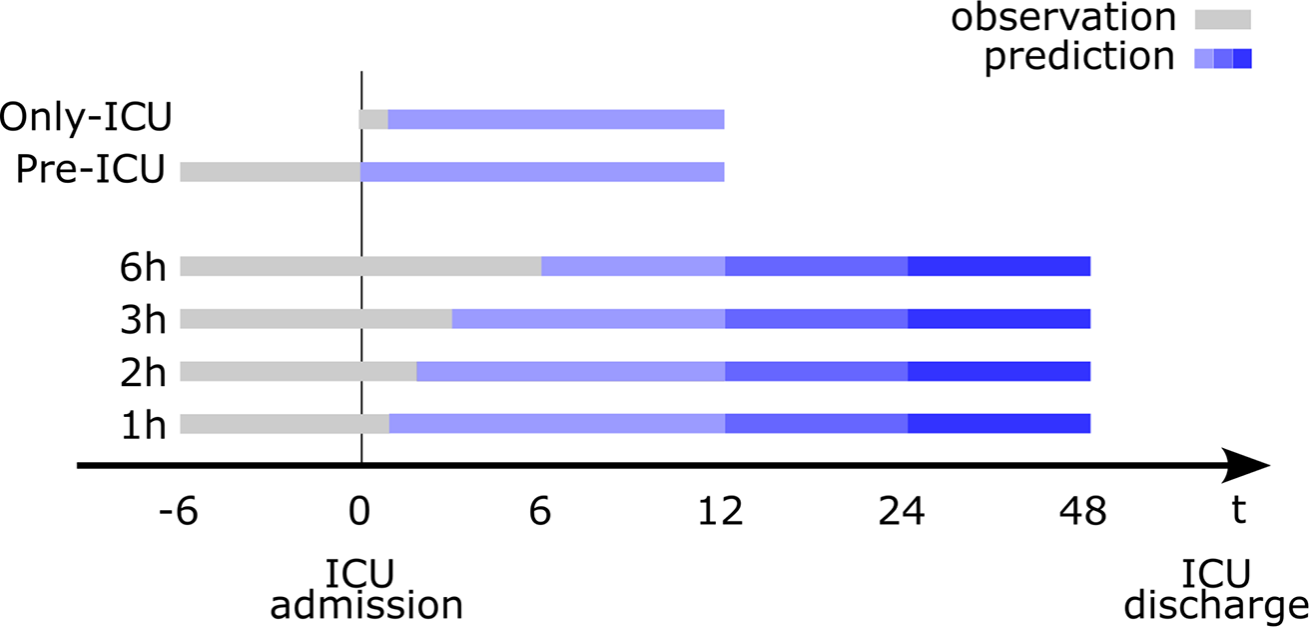
Graphical schema of the model setup. Data from the observation windows using 1, 2, 3, and 6 h after admission are used to predict a BT in the prediction windows consist of an increasing 12, 24, and 48 h after admission

The predicted outcome is defined as the first transfusion of red blood cells following admission to the ICU. Aiming to develop an effective decision support tool for the ICU, we analyzed the predictive performance improvement of our model with the incorporation of a progressively increasing amount of data as input to the model. The overall model setup is described in Fig. 1.

**Fig. 2:**
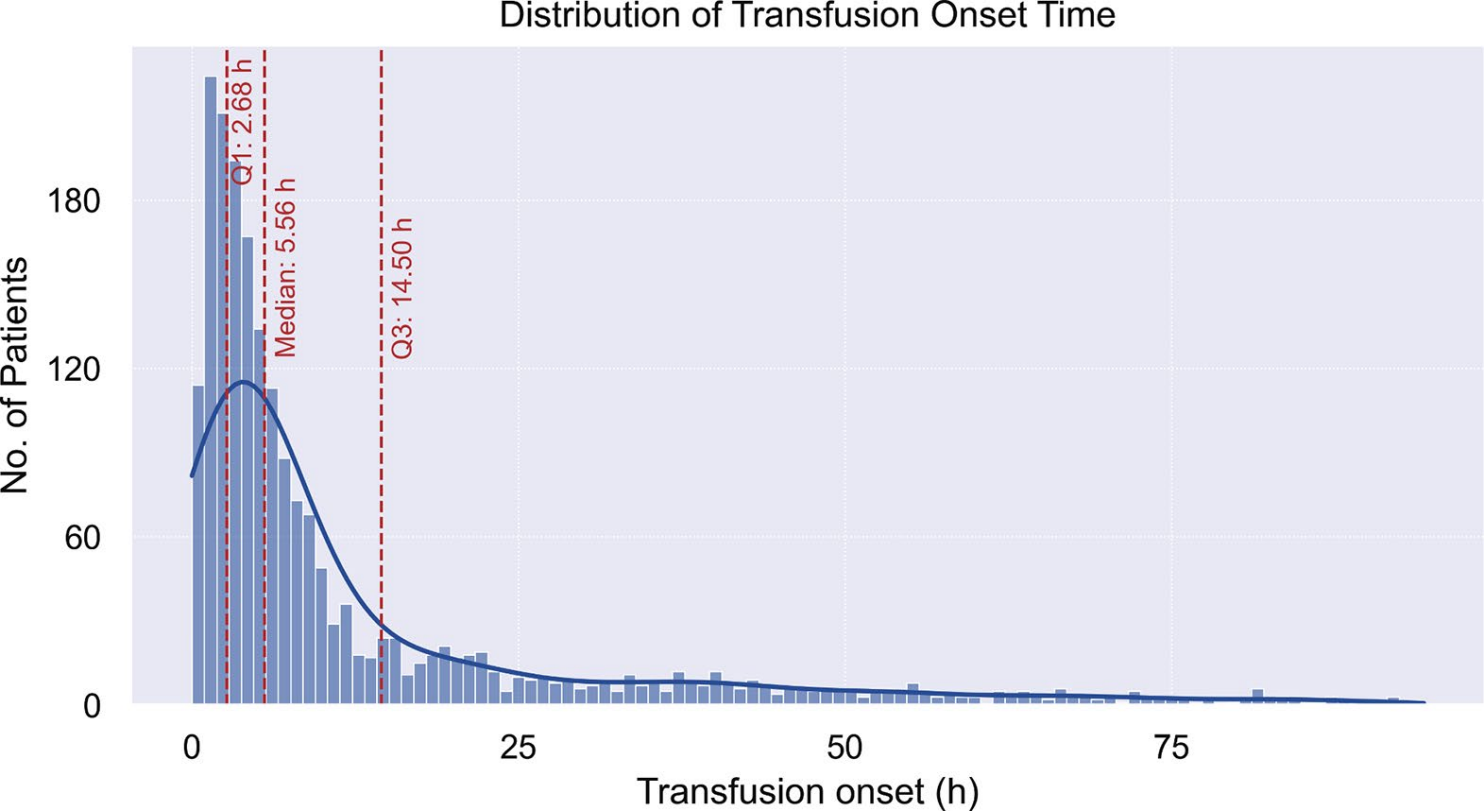
Distribution of transfusion onset time after ICU admission. The x-axis represents time in hours starting from the ICU admission and the y-axis represents the number of patients that are transfused at a specific time point. The curve shows the kernel density estimation of transfusion onset time

In the primary analysis, we included data from 6 h preceding the ICU admission up to 1, 2, 3, and 6 h following an ICU admission (observation windows), in which we selected specific data points using statistical derivations. This data was employed to predict a need for BT at intervals of 12, 24, and 48 h following ICU admission (prediction windows). These prediction windows were defined according to the actual distribution of transfusion onset following ICU admission, presented in Fig. 2. The data shows a median transfusion onset time of 5.56 h. The distribution indicates the presence of a subset of patients experiencing longer transfusion onset times compared to the majority.

The secondary analyses were conducted with aim of better understanding the impact of the different observation windows on outcome prediction: pre-ICU and only-ICU. In the pre-ICU analysis, we have a fixed observation window in which we only included data 6 h before ICU admission up to the ICU admission. In the only-ICU analysis, we only included data from ICU admission until 1 h thereafter. For both these analyses, we utilized the same prediction windows as those used in the primary analysis.

### Variable selection

We incorporated 32 clinical variables typically measured in the ICU, as identified by clinicians, that are considered relevant in predicting the requirement for BT (see Table S1 in the online supplement). These variables have been previously employed in literature and were available within the utilized dataset. Input variables include demographic data, vital sign measurement, and laboratory values.

## Data preprocessing

We implemented essential data preprocessing techniques to develop accurate predictive models. We used the Z-score method for outlier removal to enhance the data integrity. We calculated various statistical measures of the clinical variables within the observation window to capture the dynamics of the observed features. These measures included the first, minimum, maximum, and last values of the clinical variables, which are served as model input features. This approach was designed to capture a detailed and dynamic profile of clinical variables over time, thereby providing a more nuanced understanding of the trends within our observation period. Missing values were handled by imputation methods, using a multiple-imputation method for numerical variables and the most frequent method for categorical variables. To ensure fair treatment of numerical features, we scaled the data into the range of (0,1).

## Model development and evaluation

We utilized two machine learning algorithms, XGBoost (eXtreme Gradient Boosting) and LR (Logistic Regression), to predict the need for BT. XGBoost is a powerful ensemble learning method that combines multiple weak prediction models, such as decision trees, to create a robust and accurate predictive model. On the other hand, LR is a statistical modeling technique that estimates the probability of an event occurring based on input variables and serves as a baseline.

We utilized 5-fold cross-validation to train and evaluate the model, dividing the dataset into five folds. During each iteration, four folds were used for model training, and the remaining one was employed for evaluation. To address the potential class imbalance biases, we employed the StratifiedKFold method. This ensured that each fold maintained the proportion of samples for each class, avoiding biased performance evaluation.

The dataset includes an imbalanced ratio of positive and negative outcomes. Depending on the different prediction and observation windows, the positive outcome varies from 5 to 17%. This poses a major challenge for machine learning models to effectively distinguish between positive and negative outcomes. To address class imbalance in our dataset, we initially applied the class weight method to favor the minority class. However, this approach didn’t yield the desired results. We then switched to using the Youden index, which improved performance by optimally balancing sensitivity and specificity. Based on its superior effectiveness, we’ve decided to solely use the Youden index for enhancing our model’s performance.

We employed SHAP values (Shapley Additive exPlanations) to gain insights into the output generated by the XGBoost model. SHAP values offer a comprehensive interpretation of the model’s predictions by assigning importance scores to each input variable. These scores help us understand the influence of each feature on the predicted transfusion need in surgical patients, enabling us to identify the key factors driving the prediction. By employing XGBoost, LR, and SHAP values, we aimed to develop a robust predictive model while gaining valuable insights into the underlying factors contributing to a blood transfusion need.

Clinical variables are described according to the outcome using mean (± SD) or *n* (percentage), as appropriate. Mann–Whitney U-test or χ^2^ test were employed as appropriate. The predictive performance is presented using the Area Under the Receiver Operating Characteristic Curve (AUROC), the Area Under the Precision-Recall Curve (AUPRC), the Accuracy, the Sensitivity, the Specificity, Matthews Correlation Coefficient (MCC), and Brier Score. Furthermore, the Positive Rate (PR) (%) is provided as an indicator of data imbalance in each experiment.

## Results

Following the application of our inclusion criteria, a cohort of 9,118 surgical patients were selected for this study from the original UMCdb. Of those, 2,064 patients underwent a BT whereas 7,054 patients did not receive a BT. Detailed patient characteristics of these two subgroups are shown in Table 1.

While the distribution of demographic parameters appears to be consistent between both groups, most other variables had a significant difference. Patients who required BT were more likely to require mechanical ventilation, had a prolonged stays on the ICU, and a higher rate of in-hospital mortality. BT needs also vary depending on the type of surgery.

**Table 1:** Patient characteristics. Socio-demographic and encounter parameters for all included patients and divided by positive and negative outcome for UMCdb. See online supplement table S1 for other measurements

Variables	All (*n* = 9,118)	BT (*n* = 2,064)	Non-BT (*n* = 7,054)	*p*-value
Demographics				
Age, years, mean (SD)^#^	63.1 (14.5)	66.8 (13.4)	62.02 (14.6)	0.89
Female, n (%)	2,960 (32)	687 (33)	2,273 (32)	0.77
Encounter variables*				
Length of stay, hour, mean (SD)	71 (175.5)	133 (281.2)	52.8 (123.3)	< 0.05
In hospital death, n (%)	1,938 (21)	603 (29)	1,335 (18)	0.98
BT Time since admission, hours, mean (IQR)				
BT	4.9 (2.5–9.5)	4.9 (2.5–9.5)	N/A	N/A
Surgery type, n (%)				0.69
Cardiothoracic surgery	5,382 (59)	1,441 (70)	3,941 (55)	0.65
Neurosurgery	962 (10)	44 (2)	918 (13)	0.4
Gastrointestinal surgery	786 (8)	152 (7)	634 (9)	0.08
Vascular surgery	753 (8)	207 (10)	546 (7)	0.8
Other surgery	1,235 (13)	220 (10)	1,015 (14)	1
Measurements, mean (SD)				
Height, mean (SD)^#^	174.9 (9.7)	173.8 (9.5)	175.2 (9.7)	0.08
Weight, mean (SD)^#^	81.5 (15)	79 (13.7)	82 (15)	0.25
Mechanically ventilated, n (%)	7,021 (77)	1,960 (95)	5,061 (71)	0.37
Heart rate, bpm	84 (73.0–97.0)	86 (75–101)	82.0 (72.0–94.0)	< 0.05
SBP, mmHg	124 (108.0–142.0)	111 (98.0–127.0)	124 (108.0–143.0)	< 0.05
Base Excess, mmol/l	2.2 (0.8–4.4)	2.0 (0.6–4.1)	2.0 (0.8–3.8)	0.40
Ht, l/l	0.3 (0.3–0.4)	0.3 (0.3–0.3)	0.3 (0.3–0.4)	< 0.05
Hb, mmol/l	6.4 (5.7–7.3)	6.1 (5.4–6.9)	6.8 (6.0–7.7)	< 0.05
Platelets, ×10^9^/l	184 (130.0–261.0)	162 (115.0–230.0)	194 (143.0–265.0)	< 0.05
RBC, ×10^12^/l	3.6 (3.2–4.1)	3.3 (2.9–3.9)	3.8 (3.4–4.3)	< 0.05

*P*-values refer to any difference between subgroups. Abbreviations: bpm, beats per minute; BT, blood transfusion; Hb, Hemoglobin; Ht, Hematocrit; IQR, interquartile range; l, liter; mmHg, millimeters of mercury; mmol/l, millimoles per liter; n, Number; RBC, Red blood cells; SBP, Systolic Blood Pressure; SD, standard deviation

* Variables not included as input features

^#^ Mean and SD are calculated using weighted mean and weighted SD

### Feature correlation

**Fig. 3:**
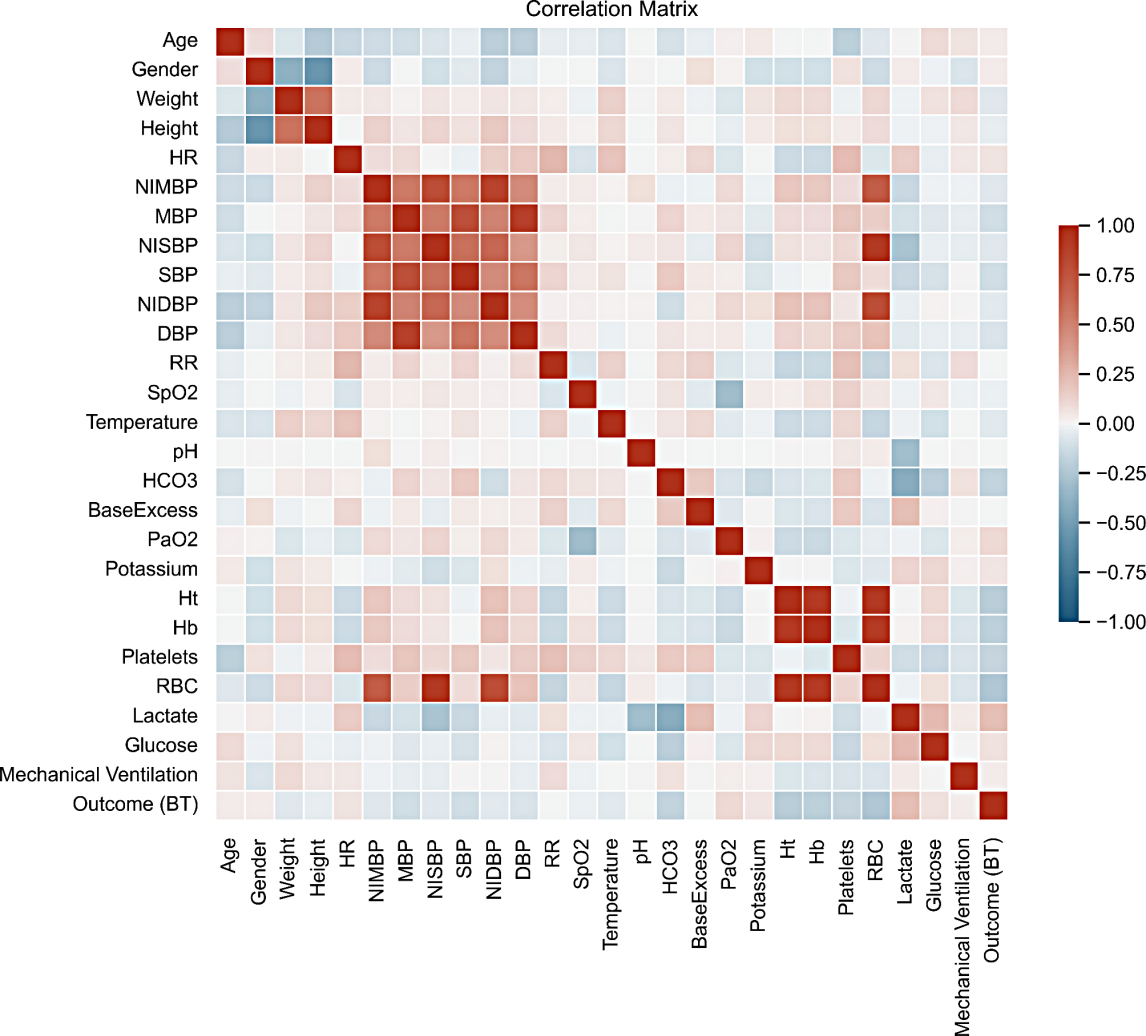
Correlations between clinical variables. The darker values are highly correlated, whereas red rectangles show a positive correlation, and blue rectangles a negative correlation

Figure 3 illustrates the correlation between all clinical variables in the overall patient selection. The strongest correlation is shown among RBC, Hb, Ht, NIMBP, NISBP, and NIDBP, which is to be expected, as different blood pressure measurements also have a strong correlation. The correlation coefficient between outcome (BT) and RBC, Lactate, Platelets, Ht, and Hb is slightly higher than any other set of variables.

### Primary analysis using pre-ICU and in-ICU data

**Fig. 4:**
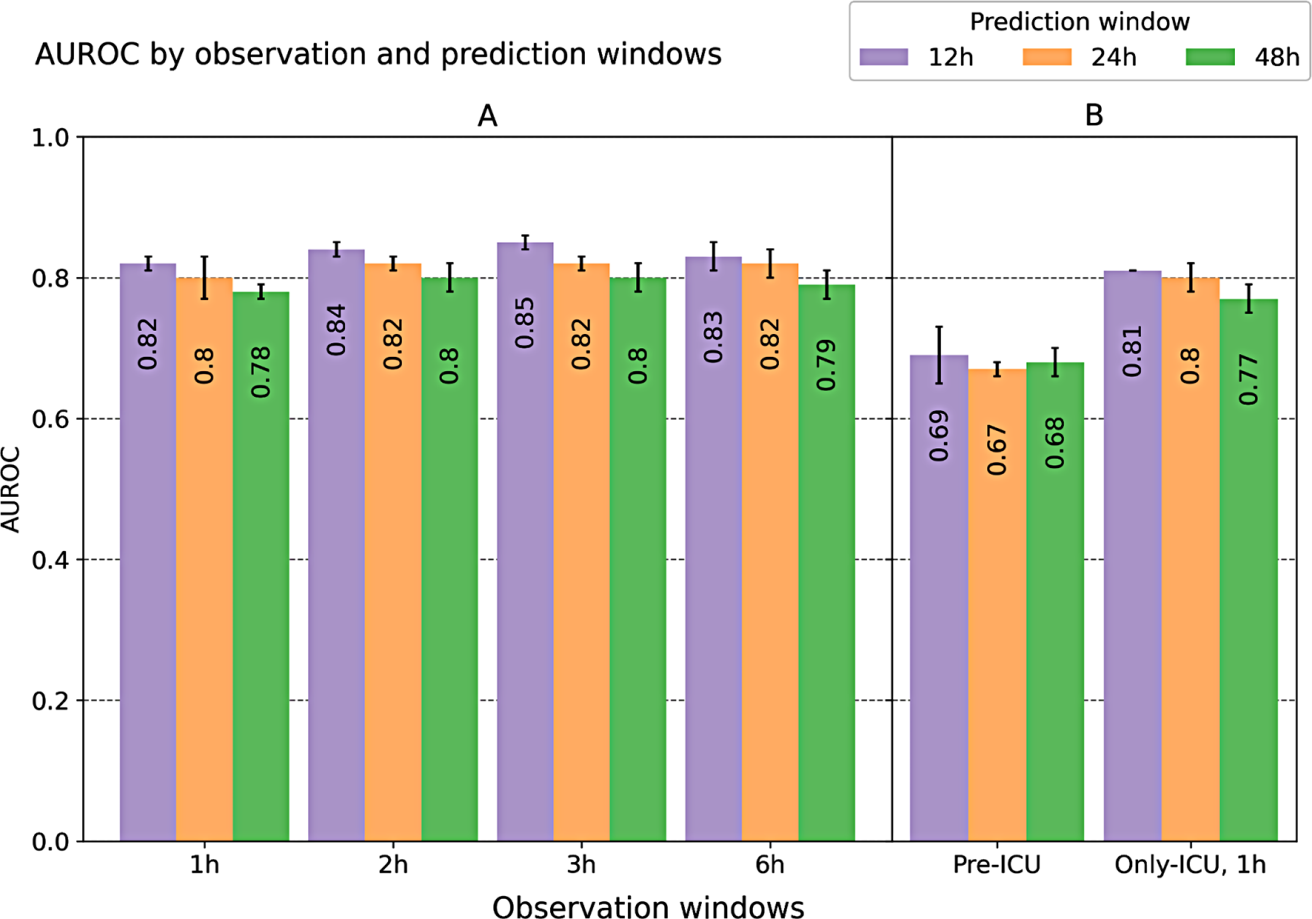
Comparison of the AUROC. Various observation and prediction windows divided by (**A**) the primary analysis and (**B**) the secondary analysis

In the primary analysis, the model input consisted of data ranging from 6 h before to an increasing period after ICU admission to predict a BT need after 12, 24 and 48 h after admission. In most experiments, XGBoost outperforms LR, but not considerably. Overall, the best performing XGBoost models target a 12-hour prediction window, as shown in Table 2. Figure 4 further illustrates the trend resulting from the AUROC analysis.

**Fig. 5:**
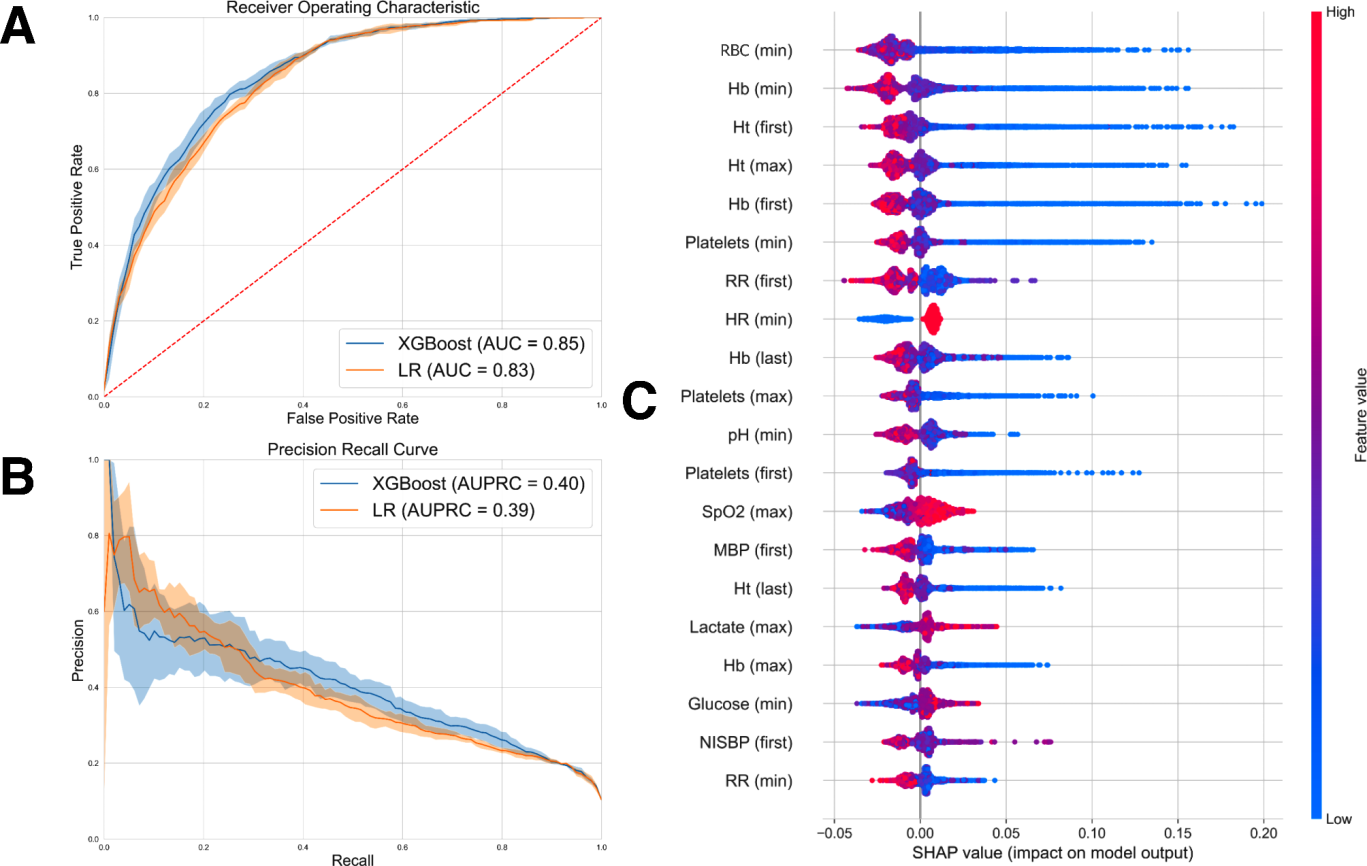
Results of the best XGBoost model. Illustrated are **A**) the AUROC, **B**) the AUPRC, and **C**) the variable ranking of the XGBoost model using SHAP algorithm reported for 3-hour observation window and 12-hour prediction window

The results from Table 2 indicates a progressive improvement in the XGBoost’s performance as the observation window extends from 1 to 3 h, likely due to increased volume of the data available over longer observation period. Interestingly, with an observation window of 6 h, the performance decreases, which is consistent with the decreasing number of BT onset. The only exception is in the observation window of 6 h with a prediction window of 12 h, where LR outperforms XGBoost with an AUROC of 0.86 ± 0.02 compared to 0.83 ± 0.02 in XGBoost. In contrast, with an increasing prediction window, the performance of the model tends to decline. This is anticipated because predicting over longer periods inherently becomes more challenging, leading to a drop in performance. These trends are similar in the other metrics, hence partially less clear. The MCC, a metric that is particularly suitable for imbalanced data and ranges from − 1 (worst) to 1 (best), yields results that correspond well with the AUROC results. Results of the best performing model are also illustrated as AUROC and AURPR in Fig. 5.

**Table 2:** Performance metrics of XGBoost model on the primary analysis. Only the 12 h prediction window is included. (OW, Observation window, PR, positive rate)

OW	AUROC	AUPRC	Accuracy	Sensitivity	Specificity	MCC	Brier Score	PR (%)
1	0.82 ± 0.01	0.40 ± 0.02	0.71 ± 0.03	0.79 ± 0.03	0.70 ± 0.04	0.35 ± 0.02	0.12 ± 0.00	13
2	0.84 ± 0.01	0.40 ± 0.01	0.72 ± 0.04	0.83 ± 0.05	0.70 ± 0.06	0.36 ± 0.02	0.11 ± 0.00	12
3	0.85 ± 0.01	0.40 ± 0.05	0.74 ± 0.03	0.83 ± 0.02	0.72 ± 0.04	0.36 ± 0.03	0.10 ± 0.00	10
6	0.83 ± 0.02	0.26 ± 0.05	0.72 ± 0.10	0.82 ± 0.08	0.71 ± 0.10	0.26 ± 0.04	0.07 ± 0.00	5

### Secondary analysis using pre-ICU or only-ICU data

Table 3 shows the performance results of the secondary analysis. If the model is based solely on the data collected after ICU admission, the performance of the models is only marginally lower compared to the primary analysis for all metrics. In contrast, if only data documented prior to the ICU admission is used, the predictability is considerably lower.

**Table 3:** Performance metrics of XGBoost model on the secondary analysis. Only the 12 h prediction window is included. (OW, Observation window, PR, positive rate)

OW	AUROC	AUPRC	Accuracy	Sensitivity	Specificity	MCC	Brier Score	PR (%)
pre- ICU	0.69 ± 0.04	0.31 ± 0.04	0.62 ± 0.12	0.68 ± 0.17	0.60 ± 0.17	0.22 ± 0.06	0.14 ± 0.00	15
only-ICU	0.81 ± 0.00	0.38 ± 0.01	0.69 ± 0.05	0.81 ± 0.07	0.67 ± 0.06	0.33 ± 0.01	0.12 ± 0.00	12

### Variable ranking

We employed the SHAP algorithm to gain valuable insights into the underlying factors contributing to transfusion requirements in surgical patients. Figure 5C ranks the top-10 variables that significantly impacted the performance of the most effective XGBoost model. In this regard, the variables that consistently emerged as influential across various analyses include Red Blood Cell count (RBC), hemoglobin (Hb), hematocrit (Ht), platelets, and heart rate (HR).

## Discussion

### Principal findings

This study investigated the application of machine learning algorithms for the prediction of red blood cell transfusions (BT) in patients admitted to the ICU after any kind of surgery. In contrast to most related work, we sought to apply BT prediction to a large cohort of patients with different surgery types, but still achieved comparably good results. We have incorporated an increasing amount of data to improve predictive performance with the increasing time the patient is on ICU. We have integrated most of the promising clinical variables from the literature and clinical expertise, with a focus on the variables typically measured in the ICU as opposed to those measured perioperatively.

Overall, the results demonstrate that conservative ML strategies can be used in a broad setting to provide healthcare professionals with early identification of patients requiring BT in the ICU.

### Related work

ML algorithms are already being applied to patients in the perioperative setting. Similar to our study, Walczak et al. [16] conducted a study using data from the American College of Surgeons National Surgical Quality Improvement Program (NSQIP) participation use files (PUF) and utilizing neural networks. Their prediction performance is slightly lower than ours. However, they do not describe the point in the patient’s stay at which the prediction is made. More studies have been conducted that also predict BT in the perioperative setting, each, however, targeting a very specific surgical procedure and are therefore not fully comparable to our study design [3, 9–11, 13]. Apart from this, all of them either have a similar or a worse performance compared to ours, although these models were created very specifically for single diseases as opposed to all kinds of surgeries in our cohort.

There are only a few related works with a focus on the ICU, which also only emphasize on internal medicine topics as opposed to surgery. For example, Levi et al. [14] proposed an ML algorithm for the prediction of rebleeding in patients with gastrointestinal (GI) bleeding as a binary classification task. Similar to our study, they trained on an increasing amount of data using observation windows of 4, 5, 6, 7, and 8 h with ongoing time. Their best performing model was trained using a 6-hour observation window and achieved an AUROC of 0.81. The most important variables were hematocrit, the amount of transfused blood, the hematocrit trend, and blood pressure. Similar to Levi et al. [14], Shung et al. [15] addressed BT in GI bleeding as a binary classification task. They proposed a deep learning algorithm to predict the first transfusion after ICU admission, with data split into 4-hour windows. They use data from one 4-hour observation window to predict a transfusion need in the subsequent 4-hour prediction window. Their best model achieved an AUROC of 0.81 and was therefore slightly worse than ours.

The only paper with a similar focus on the BT prediction on a broad set of patients, authored by Mitterecker et al. [17] using data until the end of anamnesis directly after hospital admission. Among others, they investigated the performance of four different machine learning algorithms in predicting transfusions and massive transfusions (binary classification), and the number of transfusions (regression task). The most important clinical variables in their models were hemoglobin on admission, age, diagnoses of iron deficiency and anemia, and the Charlson Comorbidity Index. They report to have achieved prediction with an AUROC of 0.96, which is much higher than the AUROC in any other related works.

The studies described above [14, 15, 17] have also translated BT prediction into a binary classification task. However, none of these studies focused on predicting BT for all surgical patients from an ICU perspective, with the amount of data increasing with increasing duration the patient stays at the ICU.

### Modeling approach

XGBoost generally exhibited a better performance than LR across various performance metrics. Nevertheless, the performance difference between the two models was not substantial. Both models showcased promising results, highlighting the potential effectiveness of machine learning for BT prediction.

Overall, the primary analysis demonstrates better performance compared to the pre-ICU and only-ICU analyses. The performance difference between pre-ICU and only-ICU reflects the different condition of the patient and the patient data at different times. The results underscore the importance of careful model evaluation and the need for context-specific adjustments when applying machine learning in the clinical setting.

We explored the impact of observation and prediction windows on the model performance. With longer observation periods, both models showed improved performance. This is likely due to the accumulation of more patient data, enabling the models to capture more meaningful patterns and trends. Conversely, as the prediction window increased, both models faced greater challenges in accurately predicting blood transfusion needs. Overly long prediction windows, however, inherently involve increased uncertainty, making the task more complex as shown with the 6-hour observation window. This finding underscores the importance of selecting appropriate observation and prediction windows when implementing machine learning models in clinical settings.

### Clinical implications

The successful application of machine learning in predicting BT for ICU patients after surgery may have several clinical implications. By identifying patients at risk of a transfusion at an early stage, appropriate measures could be initiated proactively, such as optimizing blood supply management, e.g. in terms of early blood group analysis and the possible automation of a reservation at the blood bank. It is also advantageous to be able to make predictions with as little information as possible using data from the specific department. For example, when transferring patients from surgery to the ICU, transfer errors can occur in exceptional cases, which could be compensated for with such an algorithm with regard to transfusions. A further and possibly main benefit could be knowing which patient does not require a blood transfusion. If it can be predicted that the patient will not require a transfusion and if a bed in the ICU is urgently needed for another patient, a patient could be transferred to the normal ward earlier. Of course, all the facts would have to be considered, and the prediction algorithm would have to be very reliable. Finally, such an algorithm could simply be added to the information system as a decision support system that provides alarms to staff, if necessary.

### Variable importance

The list of the most important variables included in the XGBoost model is aligned with the clinical guidelines. The hemoglobin within the red blood cells binds oxygen and carries it to the cells. The hemoglobin levels determine the blood’s oxygen-carrying capacity. When hemoglobin levels are low, named anemia, the blood’s ability to transport oxygen to tissues is reduced. Thus, hemoglobin measurements serve as a valuable indicator of a patient’s readiness for surgery and recovery progression post-surgery. Hematocrit quantifies the percentage of blood volume that comprises RBC. A decrease in hematocrit values can be indicative of anemia. Monitoring hematocrit values, therefore, provides additional confirmation of a patient’s anemic status, complementing the insights taken from hemoglobin measurements. The platelet count plays a crucial role in coagulation, hence forming an integral part of the body’s defense against excessive bleeding. A reduced platelet count could signify a heightened risk of bleeding, an important consideration in the surgical context. Lastly, heart rate serves as a direct measure of cardiac rhythm and an indicator of cardiovascular function and overall health status. An elevated heart rate might suggest physiological stress or compensatory responses to conditions such as anemia.

Each of these variables measures a unique aspect of the body’s physiological response to surgical interventions. Taken together, these variables constitute a comprehensive physiological profile that is invaluable for predicting blood transfusion risks in surgical patients.

#### Limitations and future directions

While the results are promising, this study has some limitations. First, the dataset’s retrospective nature may introduce inherent biases and limit the generalizability of the findings. Future research could involve a larger and more diverse patient cohort to validate and extend our findings. Moreover, incorporating additional clinical variables and patient-specific factors may enhance the predictive accuracy of the models further.

Causal inference aims to determine the actual effect of a particular factor or intervention on an outcome, rather than just identifying associations. This is especially relevant in clinical settings where understanding the true causal relationships can significantly influence treatment decisions and patient care strategies [19]. In this study, however, we purely focused on predictive performance, which is not equivalent to causal inference, but is sufficient for the use case.

Another limitation of our study is that we did not directly account for BTs occurred during surgeries. These intraoperative transfusions can influence the postoperative BTs. While we attempted to indirectly account for this by including data up to six hours before ICU admission, which may encompass perioperative data, this approach does not precisely isolate the impact of intraoperative transfusions. Therefore, our model might not fully capture the effects of BTs administered in the OR. This limitation highlights the need for future research to integrate comprehensive perioperative data to enhance the predictive accuracy of BTs during ICU stays.

## Conclusion

With this study, we showed the feasibility of using machine learning to predict blood transfusions (BT) in patients admitted to ICU after any kind of surgery. We also showed an increasing prediction performance with increasing data available after time the patients stay at the ICU. Challenges remain, particularly regarding the data imbalance.

## Electronic supplementary material

Below is the link to the electronic supplementary material.

Supplementary Material 1

## Data Availability

The authors will make available any data that is pertinent to this study upon a specific request, with no limitations. The analysis is based on data from the Amsterdam University Medical Centers database (UMCdb) version 1.0.2, which is accessible as described in [18].

## Abbreviations

/min: Per minute
°C: Degree Celsius
AUROC: Area under the receiver operating characteristic curve
AUPRC: Area under the precision-recall curve
bpm: Beats per minute
BT: Blood transfusion
DBP: Diastolic blood pressure
EHRs: Electronic health records
FPR: False positive rate
Hb: Hemoglobin
HCO_3_: Bicarbonate
Ht: Hematocrit
ICU: Intensive care unit
IQR: Interquartile range
L: Liter
LR: Logistic regression
MAP: Mean arterial pressure
Max: Maximum
MBP: Mean blood pressure
Min: Minimum
ML: Machine learning
mmHg: Millimeters of mercury
mmol/l: Millimoles per liter
MCC: Matthews Correlation Coefficient
MV: Mechanical ventilation
NIDBP: Non-invasive diastolic blood pressure
NIMBP: Non-invasive mean blood pressure
NISBP: Non-invasive systolic blood pressure
NPV: Negative predictive value
PaO_2_: Partial pressure of oxygen
pH: Potential hydrogen
PPV: Positive predictive value
RBC: Red blood cell
ROC: Receiver operating characteristic
RR: Respiratory rate
SBP: Systolic blood pressure
SHAP: SHapley Additive exPlanations
SD: Standard deviation
SpO_2_: Oxygen saturation
TPR: True positive rate
XGBoost: Extreme gradient boosting
