# Supplementary material for: Predicting blood transfusion demand in intensive care patients after surgery by comparative analysis of temporally extended data selection
Source: BMC Med Inform Decis Mak. 2024 Dec 18;24:397. doi: 10.1186/s12911-024-02800-z (PMC11657635; doi:10.1186/s12911-024-02800-z)
Supplement: Supplementary file 1 — Supplementary Material 1 [file 12911_2024_2800_MOESM1_ESM.pdf]

Online Supplementary Data – Predicting blood transfusion demand in intensive care patients after surgery by comparative analysis of temporally extended data selection

Table S1. Measurement characteristics. Measurement characteristics for included patients, divided by positive and negative outcome for UMCdb.

Variables	All (n=9,118)	BT (n=2,064)	Non-BT (n=7,054)	p-value
**Measurements, mean (SD)**				
NIMBP, mmHg	83 (72.0 - 94.0)	78 (67.0 - 91.0)	83 (72.0 - 95.0)	< 0.05
MBP, mmHg	80 (71.0 - 92.0)	73 (66.0 - 83.0)	81 (71.0 - 93.0)	< 0.05
NISBP, mmHg	120 (105.0 - 138.0)	113 (97.0 - 134.0)	120 (105.0 - 139.0)	< 0.05
NIDBP, mmHg	67 (58.0 - 77.0)	63 (54.0 - 74.0)	68 (58.0 - 78.0)	< 0.05
DBP, mmHg	60 (53.0 - 69.0)	56 (50.0 - 64.0)	61 (53.0 - 70.0)	< 0.05
RR, /min	18 (15.0 - 23.0)	18 (14.0 - 22.0)	18 (14.0 - 22.0)	< 0.05
SpO_2, %	97 (95.0 - 98.0)	97 (95.0 - 99.0)	97 (95.0 - 98.0)	< 0.05
Temperature, °C	37 (36.5 - 37.7)	36.7 (36.1 - 37.2)	37.0 (36.5 - 37.5)	< 0.05
pH	7.4 (7.3 - 7.4)	7.4 (7.3 - 7.4)	7.4 (7.4 - 7.4)	< 0.05
ActHCO3, mmol/l,	24.1 (22.1 -	22.5 (20.4 -	23.9 (22.1 -	< 0.05

	26.8)	24.4)	26.1)	
PaO_2, mmHg	95 (77.0 - 126.0)	109 (79.0 - 182.0)	98 (78.0 - 135.0)	< 0.05
Potassium, mmol/l	4.2 (3.9 - 4.5)	4.2 (3.9 - 4.6)	4.1 (3.9 - 4.5)	< 0.05
Lactate, mmol/l	1.3 (1.0 - 2.1)	1.8 (1.2 - 3.2)	1.3 (0.9 - 1.9)	< 0.05
Glucose, mmol/l	7.6 (6.4 - 9.0)	8.1 (6.6 - 9.7)	7.8 (6.5 - 9.3)	< 0.05

P-values refer to any difference between subgroups. Abbreviations: /min, per minute; °C, degree Celsius; BT, blood transfusion; DBP, Diastolic Blood Pressure; HCO3, Bicarbonate; MBP, Mean Blood Pressure; mmHg, millimeters of mercury; mmol/l, millimoles per liter; NIDBP, Non-Invasive Diastolic Blood Pressure; NIMBP, Non-Invasive Mean Blood Pressure; NISBP, Non-Invasive Systolic Blood Pressure; PaO_2, Partial Pressure of Oxygen; pH, potential hydrogen; RR, Respiratory Rate; SD, standard deviation; SpO_2, Oxygen Saturation.
